# Dextrin-Based Nanohydrogels for Rokitamycin Prolonged Topical Delivery

**DOI:** 10.3390/gels8080490

**Published:** 2022-08-08

**Authors:** Maria Tannous, Silvia Lucia Appleton, Gjylije Hoti, Fabrizio Caldera, Monica Argenziano, Yousef Khazaei Monfared, Adrián Matencio, Francesco Trotta, Roberta Cavalli

**Affiliations:** 1 Dipartimento di Scienza e Tecnologia del Farmaco, University of Turin, Via P. Giuria 9, 10125 Turin, Italy; 2 Dipartimento di Chimica, Università di Torino, Via P. Giuria 7, 10125 Torino, Italy

**Keywords:** rokitamycin, cyclodextrin, linecaps, nanosponges, formulation, nanohydrogels

## Abstract

Macrolides are widely used antibiotics with a broad spectrum of activity. The development of drug carriers to deliver this type of antibiotics has attracted much research. The present study aims at developing new swellable dextrin-based nanohydrogels for the topical delivery of rokitamycin, as model macrolide. Rokitamycin is a synthetic analogous of macrolides with advantageous characteristics as far as bacterial uptake and post-antibiotic effect are concerned. It is also indicated for the treatment of severe infections caused by *Acanthamoeba* and for topical infections. The nanohydrogels have been prepared from two types of cross-linked polymers obtained by using β-cyclodextrin or Linecaps^®^ was provided by the Roquette Italia SPA (Cassano Spinola, Al, Italy) as building blocks. The cross-linked polymers have been then formulated into aqueous nanosuspensions refined and tuned to achieve the incorporation of the drug. Cross-linked β-cyclodextrin (β-CD) and Linecaps^®^ (LC) polymers formed dextrin-based nanohydrogels with high swelling degree and mucoadhesion capability. Rokitamycin was loaded into the nanohydrogels displaying an average size around 200 nm with negative surface charge. In vitro kinetic profiles of free and loaded drug in nanohydrogels were compared at two pH levels. Interestingly, a sustained and controlled release was obtained at skin pH level due to the high degree of swelling and a pH responsiveness possibly. The results collected suggest that these nanohydrogels are promising for the delivery of rokitamycin and may pave the way for the topical delivery of other macrolide antibiotics.

## 1. Introduction

Antibiotic resistance is one of the biggest health problems today [1]. Bacteria have progressively gained resistance to the therapeutic agents used against them, antibiotics. Among all, macrolides are effective on Gram-positive and Gram-negative bacteria, including *Actinomycetes* and *Mycobacteria*, as well as *Treponemes, Mycoplasmas, Chlamydiae, Rickettsiae*, and some *Protozoa* [1,2,3] by inhibiting protein synthesis. In detail, they selectively block the translation of a subset of cellular proteins by antibiotic structure, being recently defined as “modulators of translation”, being a promising drugs against antibiotic resistance [4]. Alongside the synthesis of new macrolide derivatives to improve the bioavailability and antibacterial properties, new delivery systems have been investigated for their administration [5]. Interestingly the delivery of antibiotics using nanocarriers is a promising strategy to overcome drug resistance [6]. Drug nanocarriers, having nanoscale size can improve the pharmacokinetic of a free drug achieving a prolonged kinetics to extend the resistance time and can be designed to be uptaken by the cells.

In this study Rokitamycin (RK) was selected as a model macrolide antibiotic.

**Figure 1:**
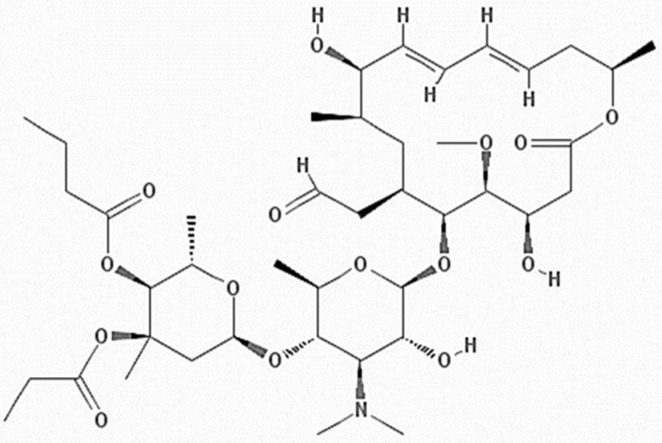
Chemical structure of Rokitamycin (C_42_H_69_NO_15_).

RK (Figure 1) is a semisynthetic 16-membered ring macrolide, which has been studied as an alternative to erythromycin to treat antibiotic-susceptible and/or resistant bacteria [3]. Unlike other macrolides, it is more hydrophobic, has better bacterial uptake, more cohesive ribosomal binding, and a longer post-antibiotic effect. In addition, it was found to induce cell death in a dose-dependent manner [7]. Moreover, it has also been associated with cutaneous lesions, arthritis, and sinusitis in AIDS patients and other immuno-compromised patients [8].

However, its low bioavailability, unpredictable pharmacokinetics, and low stability at acid pHs (e.g., in the stomach) make its formulation challenging [9]. Previously, several approaches have been made to formulate RK in order to improve its water solubility, antiamoebic effect in vitro, and reach the target in vivo. For example, Rassu et al. [3] develop chitosan microspheres to load RK and achieve a controlled release or the evaluation of *Acanthamoeba castellani* growth rate when treated with the RK-loaded formulation showing that the carrier selected was able to load and prolong the drug effect. A subsequent study [10] aimed at optimizing the chitosan microsphere formulation method by testing chitosan derivatives and positive results were achieved as far as size, in vitro release, and mucoadhesion were concerned, thus may make them more suitable for ocular and nasal administration.

Nevertheless, antibiotic resistance needs to be studied from a deeper point of view, in particular the possibility to get combinatorial effects between drugs and excipients/carrier, as occurs with Nanosponges (NSs), which are hyper-crosslinked polymers which can be also considered nanhydrogels [11] when suitable cross-linking agents are used.

They are particularly promising in the pharmaceutical field as nanosized drug delivery systems [12]. They are biocompatible, biodegradable, and their production is flexible and cheap due to their simple synthesis and purification as well as the use of a limited number of reagents [12,13,14,15,16].

Their peculiar structure, generally made up of cyclodextrin units crosslinked by crosslinking agents, enabled different kinds of drugs to be encapsulated due to the formation of inclusion and non-inclusion complexes, which is attributable to the availability of high number of interaction sites, i.e., hydrophobic cyclodextrin cavities and hydrophilic nanochannels of the polymeric network [17,18,19,20,21]. Moreover they showed sustained drug released kinetics, which is a desired property for improving drug bioavailability and effectiveness [22,23,24].

Nanosponge polymers have been employed for the delivery of antibiotics only in a few studies [25,26]. Recently, the antimicrobial capacities of the NSs generated an interesting combinatorial effect with the antimicrobial peptide Nisin [24]. This last point suggests that the antimicrobial activity of RK might be increased not only by increased stability and better bioavailability [25], but also by the intrinsic antimicrobial capacity of the excipient. Moreover, other drugs such as norfloxacin, for example, were loaded in NSs to achieve an oral administration with improved intestinal absorption [26]. In another work, NSs were impregnated with lysozyme in order to break bacterial cell walls and NSs themselves were proposed as an alternative to avoid or reduce toxic effects and resistance to therapeutics due to their intrinsic antimicrobial action [27].

Everything said above suggests that the vehiculation of RK with a NS carrier might have an important therapeutic topical application. This work aimed at investigating two dextrin-based nanohydrogels as topical delivery systems for Rokitamycin. The nanohydrogels will be obtained exploiting two different building blocks, i.e., β-cyclodextrin or Linecaps^®^, and using pyromellitic dianhydride (PYRO) as cross-linking agent.

**Figure 2:**
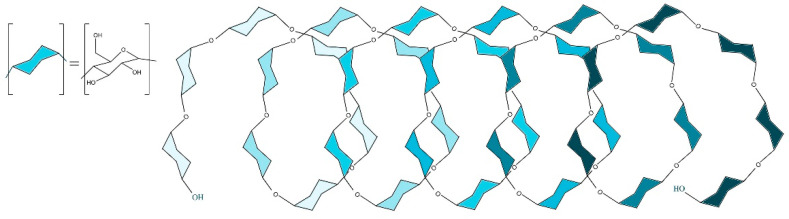
Helical structure of amylose Linecaps^®^.

Due to its origin from pea starch, Linecaps^®^ is rich in linear and soluble amylose. This amylose fraction enable the formation of inclusion compounds as previously shown for taste masking and other applications. Amylose exists as helical structures, and like a cyclodextrin these bear a hydrophilic external surface and a hydrophobic internal cavity, created by the presence of glycosidic ether bonds [28]. Linecaps^®^ is a dextrin derivative with a helix structure as shown below (Figure 2).

The present study focused on an in vitro approach to optimize the RK- nanohydrogel formulations, in particular the work will consider: (i) A complete physico-chemical characterization using different techniques (FTIR, TGA, DSC, SEM); (ii) the evaluation of the encapsulation efficiency and drug loading; (iii) the control of the swelling degree, viscosity, and mucoadhesion; and (iv) the release kinetics study.

## 2. Results and Discussion

The use of nanohydrogels for the delivery of lipophilic molecules has been previously proposed. Different approaches have been investigated to incorporate hydrophobic drugs in a polymer matrix.

The presence of cyclodextrin units within the nanogel structure can promote the hydrophobic drug incorporation [24,29,30,31,32]. β-CD nanosponges (NSs) are chemically crosslinked polymers that have many attractive features for use as hydrogels.

**Figure 3:**
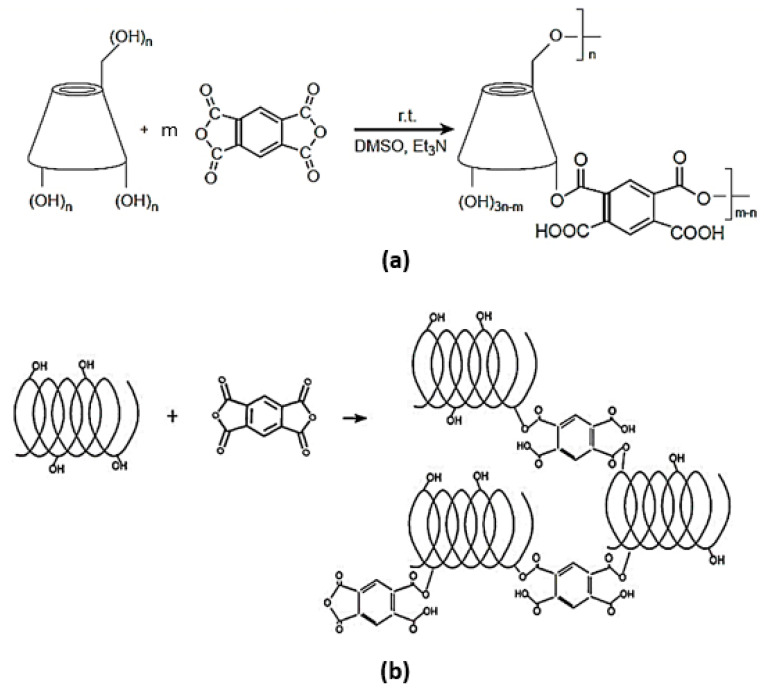
Scheme of the synthetic procedures to obtain (**a**) β-CD PYRO and (**b**) LC PYRO NSs.

At first it is important to underline that in this work two types of swellable NSs or nanohydrogels were prepared, starting from two different building units namely β-CD and LC (cyclic for CD and linear for LC). β-CD PYRO and LC PYRO NSs were synthetized using pyromellitic dianhydride as cross-linking agent at molar ratio 1:4 (Figure 3). The calculated molecular weights of Linecaps^®^ are not a true representation for the maltodextrin, as Linecaps^®^ has a broad molecular weight distribution. Therefore, the Linecaps NS was prepared, following the synthesis method of β-CD PYRO NS, by simply replacing the amount of β-CD with an equivalent weight of Linecaps^®^. As a result, the two NSs have approximately the same molar ratio of glucose units to linker (i.e., 7:4) and therefore a similar degree of cross-linking.

To our best knowledge, LC PYRO NSs have been here described for the first time.

The structure of NSs is strongly dependent on the type of the cross-linker chosen. The cross-linking reaction of dianhydrides with OH groups is widely discussed in the literature. Dianhydrides are suitable cross-linkers due to their reactivity with nucleophiles, such as the OH groups of β-CD and LC structure. The presence of free carboxylic groups in the polymer network produces a swellable polymer matrix. This unique property of the NSs here synthesized enables swelling in the biological and aqueous environment [33,34,35].

**Figure 4:**
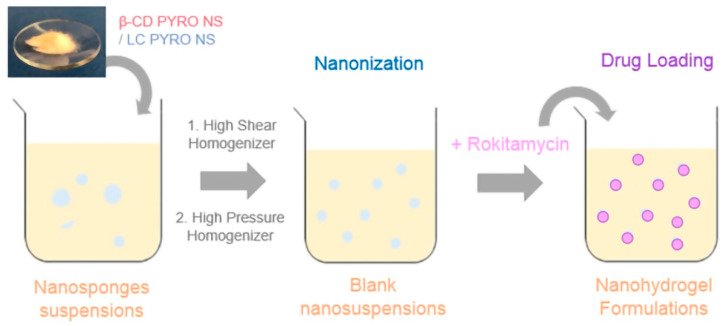
Schematic representation of the nanohydrogel formulation.

The formed polymer matrices, named β-CD PYRO NSs and LC PYRO NSs, were subsequently suspended in aqueous solutions and underwent a nanosizing process using the high pressure homogenization (HPH) technique, to obtain the nanohydrogels (Figure 4).

### 2.1. Physicochemical Characterization and Loading of Nanohydrogels

Different parameters such as the particle size and Z-potential were evaluated to characterize the unloaded and loaded nanohydrogels and to understand if the RK incorporation changed their values. The average particle size of the blank NSs β-CD PYRO and LC PYRO and RK-loaded nanohydrogels in aqueous nanosuspensions was around 200 nm with polydispersity indices lower than 0.1. (Table 1). Stability studies were performed on the NSs after one month stored at 4 °C (Table 2).

The low polydispersity index values show a uniform size distribution within the nanoparticle population due to the HPH process. Both nanohydrogels showed negative surface charges suitable for avoiding the nanosponge aggregation phenomenon, thus indicating their ability to form physically stable nanosuspensions in an aqueous medium. After loading with RK, a slight increase (about 15%) of the particle size was observed together with the decrease of zeta potential values which could be attributable to the interaction between RK and NSs and the formation of the drug complex [36].

No significant changes in the physico-chemical parameters of the two nanohydrogels were observed after 1 month, confirming the physical stability of the nanoformulations stored at 4 °C. Another parameter measured was the viscosity, which is important to determinate the possible alteration of rheologic properties of the formulation. It showed for β-CD PYRO and LC PYRO NSs aqueous suspensions a value of 0.941 cP and 1.045 cP respectively that did not change over time.

The nanohydrogels showed a valuable mucoadhesion capability (Table 1). After the RK incorporation, a decrease of about 10% in the mucoadhesion percentages was observed, reaching the 76.93 and 72.39% for β-CD PYRO and LC PYRO NSs, respectively. We can speculate that these values would be still suitable to assure mucoadhesion after topical application and the complexation does not affect the mucoadhesion in a high proportion [18].

The HPLC analysis of the loaded formulations showed a loading capacity of RK in β-CD PYRO of 8.02 ± 0.51% and 8.50 ± 0.45% for LC PYRO NSs while the encapsulation efficiency calculated was 88.62 ± 1.05% and 89.71 ± 0.86% for β-CD PYRO and LC PYRO NSs, respectively. These values are in accordance with previous drugs complexes such as stilbenes or kynurenic acid for other types of NSs [29,30].

The RK solubility enhancement after the NS incorporation was then investigated. Saturation solubility tests were performed on both β-CD and LC NSs. Absorbance spectra were collected and the RK solubility was determined using an external calibration method. The solubility was enhanced 3.1 fold in case of LC and 1.2 fold for β-CD complexes at pH 5.5 (Table 3).

It could be postulated that the enhanced apparent solubility of RK is due to the NS incorporation and masking of hydrophobic groups of rokitamycin by nanosponge matrix [37].

### 2.2. Swelling Capacity

The capacity to absorb water of the NS was recently reported showing values up to 1000% in some cases, this capacity lets the material to form gels with interesting properties [34]. Interestingly both β-CD and LC NSs were easily able to absorb water-forming nanohydrogels, and their swelling capacity was investigated on freeze-dried samples as a function of the pH value of the external environment. Both materials showed a marked swelling capacity related to the cross-linking degree, which depends on the environmental pH value. Similarly, both nanohydrogels were pH sensitive due to the presence of dissociable carboxylic groups in the polymer matrices. Indeed, increasing the pH the carboxylic groups underwent deprotonation with subsequent modification of the polymer matrix due to repulsive forces which cause the enhanced swelling degree. This behavior is in agreement with previous results obtained with β-CD PYRO nanosponges [18].

The swelling degree of free and RK-loaded NSs at pH 5.5 and pH 7.4 is shown in Table 4. LC-based nanohydrogels showed a greater swelling capability than β-CD nanohydrogels.

The water uptake capability is high in both nanohydrogels (Table 4). The LC PYRO nanohydrogel showed a greater swelling degree at both pH values compared to β-CD PYRO nanohydrogel. This difference can be related to the different polymer matrices of the two nanohydrogels, one containing β-CD and the other LC as a building block causing a different cross-linking ratio.

Our recent publication demonstrated that different cross-linking densities could affect swelling. In this case, although the crosslinker ratio is the same, the different structure of monomers (cyclic for CD and linear for LC) and molecular weight could affect in a similar way, justifying the differences [34]. The incorporation of RK in NSs slightly affected their swelling degrees.

### 2.3. Structural Analysis: Fourier Transformed Infrared and Differential Scanning Calorimetry

**Figure 5:**
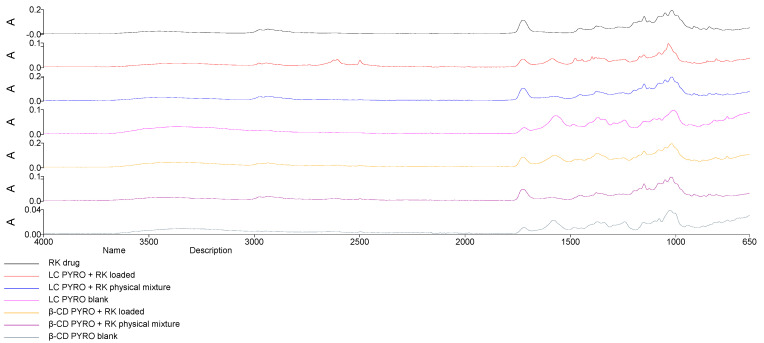
FTIR analysis of RK, blank NSs, physical mixtures, and loaded NSs indicated the principal differences.

The FTIR analysis in Figure 5 showed characteristic peaks of RK around 3550 cm^−1^ (phenol O–H stretching), 2985 cm^−1^ (alkyl C–H stretching), 1750 cm^−1^ (ester C=O stretching), and peaks at around 1250 cm^−1^. The spectra of RK-loaded NSs unlike that of physical mixtures (RK and NSs) does not show the characteristic peak of RK (band at 1750 cm^−1^) and band 3550 cm^−1^ is broader and shifted to higher wavelength suggesting the formation of hydrogen bonds that might indicate inclusion within the polymer cavity. The disappearance and the shift of peaks suggest interactions between RK and NSs.

**Figure 6:**
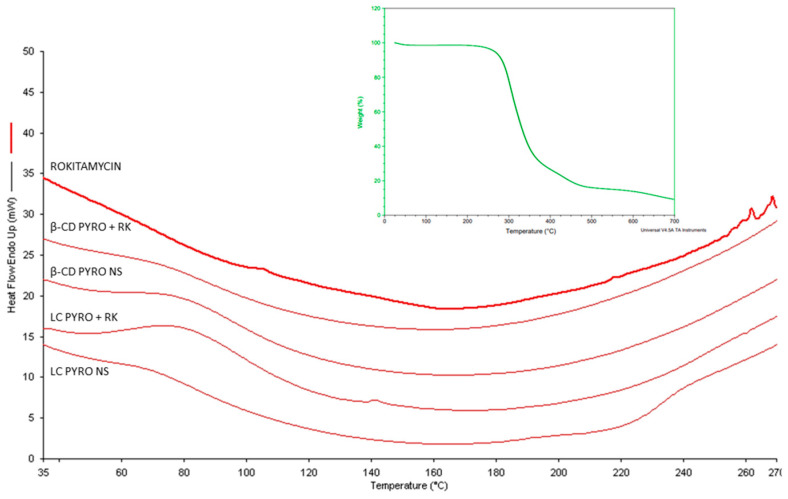
DSC thermograms RK, blank NSs, and RK-loaded NSs. Insert. TGA thermogram of Rokitamycin.

In addition, DSC analysis (Figure 6) proved the incorporation of RK within the two polymer matrices. Interestingly, the melting peak of RK at about 260 °C as previous TGA thermograms indicated the same melting point RK (Figure 6 insert). The disappearance of RK peaks suggests the molecular interaction of the drug with the polymer matrices. Finally, the TGA of polymers (data not showed) at pH 5.5 and 7.4 presented differences in water absorption in accordance with the swelling capacities previously reported. This thermal behavior suggests that the drug is molecularly dispersed inside the nanostructure of NSs and was not able to crystallize, in agreement with other NS formulations [38].

### 2.4. Morphology Analysis: Scanning Electron Microscopy

**Figure 7:**
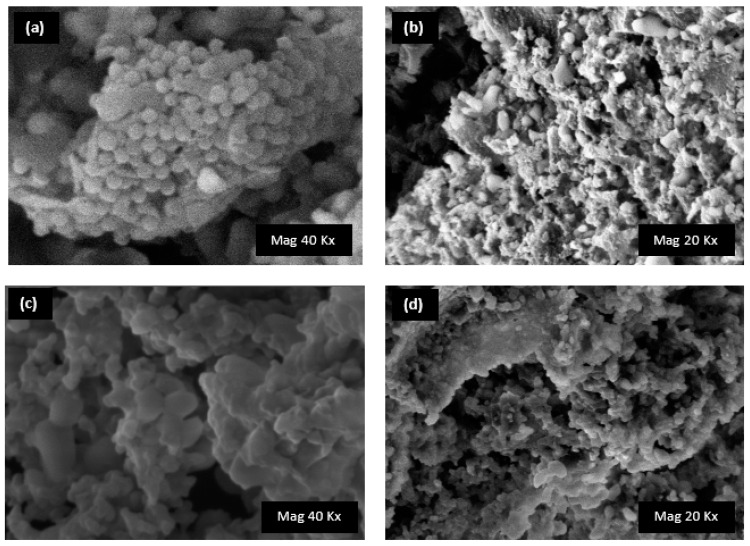
SEM images of (**a**) blank β-CD PYRO (1:4), (**b**) RK-loaded β-CD PYRO (1:4), (**c**) blank LC PYRO (1:4), and (**d**) RK-loaded LC PYRO (1:4).

The morphology of the two nanogels was evaluated by SEM analysis. Due to the presence of β-CD or LC as monomers in the synthetic procedure, differences were observed between the two samples. Noticeably, the SEM image of the unloaded β-CD PYRO NS showed spherical nanoparticles, while aggregates with smooth surface were observed for LC PYRO NS [39]. The SEM images of RK-loaded nanoformulations (Figure 7) did not present marked modification on the nanostructure, confirming the drug incorporation. These results were in agreement with the physico-chemical parameters (average diameter an Z-potential (Table 2)) [40].

### 2.5. Rokitamycin Release Studies

**Figure 8:**
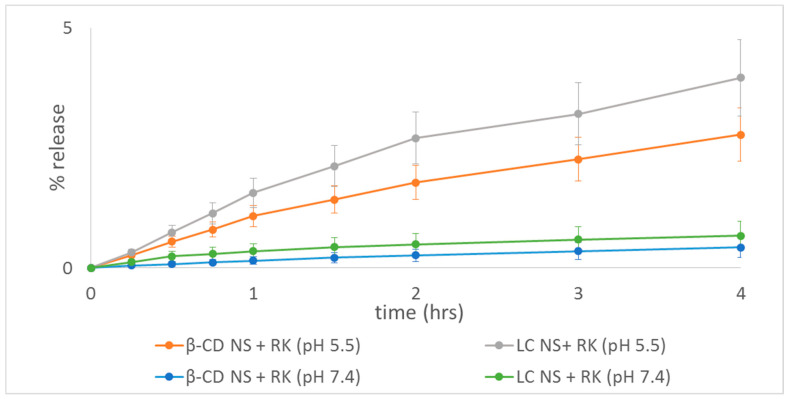
In vitro release profiles of RK-loaded NSs in pH 5.5–7.4 the first 4 h.

RK is a drug with poor water solubility and its solubility increases with a reduction of the pH of the receiving media due to its gradual protonation [41]. The solubility of the drug in phosphate buffer pH 6.5 was previously determined [3]. Here we investigated the RK release kinetics at pH 5.5 and 7.4, we selected pH 5.5 to simulate the skin pH value [42]. Figure 8 shows that the pH value certainly affected the diffusion kinetics of RK. Interestingly, both NSs improved the release profile of RK at pH 5.5 compared to pH 7.4. At pH 7.4 RK being not protonated, is more lipophilic and has a lower water solubility. No initial burst effect was observed and RK was released gradually overtime from both nanogels. No significative differences were observed between both polymers, although LC seems to release faster RK, according to our previous solubility increase results.

These data support additionally the idea that RK was successfully loaded inside NS polymer matrix and not weakly adsorbed onto the NS surfaces, improved its solubility and released it in a sustained manner. The prolonged release of the drug, extending the residence time, might create a higher formulation activity.

This slow release kinetics can be related to the slow diffusion of the drug within the swelled polymer matrix under these experimental conditions as previously proven by cyclodextrin-based nanohydrogels [19,42,43].

**Figure 9:**
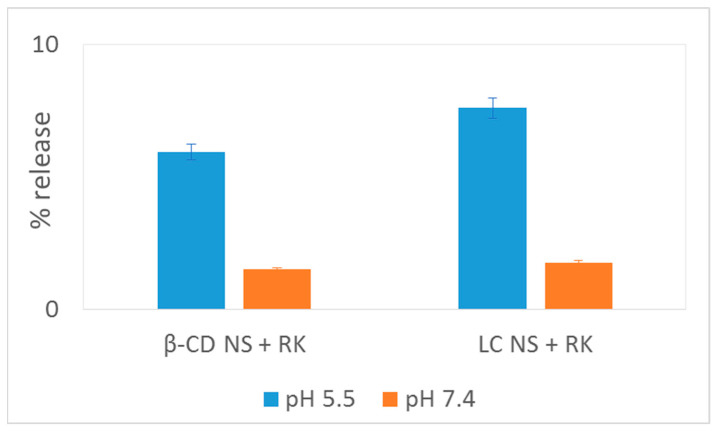
Release of RK-loaded NSs in pH 5.5–7.4 after 28 h.

The RK release was around 7.6% and 5.9% at pH 5.5 after 28 h for LC and β-CD respectively (Figure 9). The RK release is affected by the swelling degree of the nanohydrogels. Remarkably, the release kinetics is faster at the skin pH value.

### 2.6. Mathmatical Model Fitting

The mechanism of drug release was evaluated applying selected mathematical models (Table 5).

The release constant was calculated from the slope of the appropriate plots, and the regression coefficient (r^2^) was determined. The release kinetics of RK from β-CD PYRO NS exhibited great correlation with the Korsmeyer–Peppas kinetics model (r^2^ = 0.9988), suggesting that the release from NS is influenced by the drug diffusion from a swellable polymer matrix [42]. Higuchi’s model describes the release of drugs from an insoluble matrix as a square root of a time-dependent process based on Fickian diffusion. The release of RK from LC PYRO NS was best associated by Higuchi’s equation as the plots showed high linearity range (r^2^ = 0.9951). This explains why the drug diffuses at a comparatively slower rate as the distance for diffusion increases, which is referred to as square root kinetics.

### 2.7. Evaluation of Biocompatibility

Red blood cell (RBC) hemolysis was performed to assess the formulation’s biocompatibility. Negligible hemolysis was observed for β-CD and LC samples at the concentrations used in the biological assays, which confirms the formulations biocompatibility. These results are consistent with the literature [13].

## 3. Conclusions

This study investigated two different swellable nanohydrogels as an interesting nanotechnological approach to the local delivery of macrolides after topical administration, using RK as a case study due to its relevance in the treatment of severe infections. The two kinds of nanohydrogels selected for this purpose, PYRO β-CD and LC-based NSs were not only able to encapsulate RK, thus improving its low solubility in water and releasing it in a sustained way, but also showed a remarked mucoadheshion compatibility. The interaction between RK and nanohydrogel matrices was further confirmed by FTIR and DSC analyses. These preliminary results suggest that these two types of nanohydrogels may be suitable for topical delivery of RK and encourage future studies to assess the efficacy of RK loaded with the two nanoformulations.

## 4. Materials and Methods

### 4.1. Chemicals and Reagents

β-CD was provided by Roquette Frères SA (Lestrem, France). Linecaps^®^ (LC) was kindly supplied by Roquette Italia (Cassano Spinola, Italy). Both were dried in oven at 80 °C overnight before use. RK (RK) was kindly given by Grunenthal Formenti Laboratories (Milan, Italy). Dimethylsulfoxide (DMSO), trimethylamine (TEA), pyromellitic dianhydride (PYRO) were purchased from Sigma-Adrich (Munich, Germany). Deionized and milliQ^®^ water were obtained using a Millipore Direct-QTM 5 production system. All other chemicals were commercially available.

### 4.2. Synthesis of Dextrin-Based Nanosponges

To obtain the nanohydrogels, two types of nanosponges have been prepared according to a previous protocol with slight modifications [16]:

- β-CD PYRO(1:4) NSs was prepared as follows: 14.10 g of dehydrated β-CD was added to 16.6 mL of DMSO inside a round bottom flask and left stirring until a colorless homogeneous solution is obtained. TEA (4.17 mL) was then pipetted into the flask followed by addition of 3.13 g of PYRO. At the end of the process a dark amorphous solid-like gel was formed and left overnight to solidify. The resulting nanosponge was removed and ground in a mortar and pestle and then washed in a Buchner with 500 mL of distilled water, and 500 mL of acetone. Once again it was left to dry and then extracted with acetone in a Soxhlet at 60–70 °C for 48 h. Finally, the coarse powder was packed and stored in a dry medium.
- LC PYRO (1:4) NSs: Inside a dry clean round bottom flask, 9.78 g of LC was solubilized under continuous stirring in 40 mL of DMSO and then 10 mL of TEA and 7.55 g of PYRO were added. The molar ratio used between Linecaps and cross-linker was 1:0.57, expressed as molar ratio of one mole of condensed glucose of maltodextrin (molar mass of 162.15 g/mol) with respect to 0.57 moles of cross-linker. After 24 h, the reaction was complete and the nanosponges were ground and washed with deionized water in a Buchner funnel and then left for drying. The purification was carried out by means of Soxhlet extraction with acetone for 14 h.

### 4.3. Preparation of Blank Nanosuspensions

β-CD PYRO (1:4) NSs and LC PYRO (1:4) NSs were suspended in saline solution (NaCl 0.9% *w*/*v*) at a concentration 10 mg/mL by means of high shear homogenizer (Ultraturrax^®^, IKA, Konigswinter, Germany) for 10 min, and then subjected to high pressure homogenization (HPH) for 90 min at a back-pressure of 500 bar (EmulsiFlex C5, Avastin, Ottawa, ON, Canada). After that the pH was corrected to 5.5 using NaOH solution (0.2 M) and then the nanosuspensions were purified by dialysis to eliminate any residues (Spectrapore, cellulose membrane, cutoff 12,000 Da) and then stored at 4 °C. Later on, freeze-drying was done to obtain a dry powder.

### 4.4. Rokitamycin Loading Procedure

RK was loaded by adding a weighed amount of RK to blank nanosuspensions at a concentration 1.5 mg/mL and left stirring at room temperature away from light for 24 h. Centrifugation was performed (10,000 rpm, 10 min) to separate the free RK from the loaded nanosuspensions. Then the nanoformulations were freeze-dried overnight. The amount of RK complexed inside NSs was determined via HPLC-UV analysis.

RK loaded in the nanosuspensions was incubated for 1 h at room temperature and then dialysis was done to release free RK. A known volume of each complex was centrifuged in micropore Eppendorf (20,000 rpm for 5 min) and diluted and analyzed via HPLC-UV method.

### 4.5. Physicochemical Characterizations

#### 4.5.1. Dynamic Light Scattering (DLS)

A 90 plus particle sizer DLS (Brookhaven Instruments Corporation, Wakefield, MA, USA) was used to determine the average diameter and polydispersity index of the nanoformulations. The measurements were set at a fixed scattering angle of 90 and at 25 °C. The same instrument was used to measure the zeta potential by applying an electric field of approximately 15 V/cm.

#### 4.5.2. Fourier Transformed Infrared (FTIR)

Perkin Elmer Spectrum 100 FTIR was used to analyze FTIR spectra of RK, blank NSs, and RK-loaded NSs in the region of 4000–650 cm^−1^ in the attenuated total reflectance ATR mode with a diamond crystal, using 32 scans per spectrum and a resolution of 4 cm^−1^. Data acquisition was done with spectrum software version 10.03.05 Perkin Elmer Corporation.

#### 4.5.3. Thermogravimetric Analysis (TGA)

TGA analysis was performed in a TA Instrument TGA Q500 (New Castle, DE, USA). Approximately, 10 mg of lyophilized NSs was weighed in an alumina pan and heated at 10 °C/min from room temperature to 700 °C, under nitrogen flow. The thermograms were elaborated using TA Instruments Universal Analysis 2000 software (version 4.5A) (New Castle, DE, USA).

#### 4.5.4. Differential Scanning Calorimetry (DSC)

DSC was carried out by means of a Perkin Elmer DSC/7 differential scanning calorimeter (Perkin-Elmer, Shelton, CT, USA) equipped with a TAC 7/DX instrument controller. The instrument was calibrated with indium for melting point and heat of fusion. A heating rate of 10 °C/min was employed in the 25–250 °C temperature range. Standard aluminum sample pans (Perkin-Elmer) were used; an empty pan was used as a reference standard. Analyses were performed in triplicate on 3 mg samples under nitrogen purge.

#### 4.5.5. Scanning Electron Microscopy (SEM)

The surface morphology of β-CD and LC nanohydrogels lyophilized powders free and loaded with RK was determined by scanning electron microscopy (SEM) using a Leica Stereoscan410 (Wetzlar, Germany). The samples were observed after Au metallization. The voltage used was in the 5 kV to 10 kV range.

#### 4.5.6. High Performance Liquid Chromatography (HPLC)

RK was analyzed using an HPLC pump (Perkin Elmer 250B, Waltham, MA, USA) equipped with a spectrophotometer detector (Flexar UV/Vis LC, Perkin Elmer, Waltham, MA, USA). The analytical column selected was a C18 (250 mm × 4.6 mm, ODS ultrasphere 5 μm; Beckman Instruments, Brea, CA, USA). The mobile phase consisted of a mixture of acetonitrile and ammonium acetate solution 0.05 M (60:40 *v*/*v*) at pH 7.0 with 1 mL/min flow rate. UV detection was set at lambda 230 nm.

RK stock solution was prepared by dissolving RK in acetonitrile. It was diluted with mobile phase to prepare calibration levels, which were injected into the HPLC. Linear calibration curves were obtained over the concentration range of 5−85 μg/mL with regression coefficient of 0.999 and were used to determine RK concentration.

### 4.6. Encapsulation Efficiency and Loading Capacity

RK-loaded NSs were dispersed in mobile phase and subjected to sonication for disruption of the complex and release RK. The amount of RK was determined by HPLC and encapsulation efficiency and loading capacity were calculated following specific formulas [44]. The experiments were performed in triplicate and the results are expressed as mean ± standard error.

### 4.7. Mucoadhesion Capability Evaluation

The interaction of mucin and NSs (both blank and RK-loaded) was evaluated via turbidimetric measurements. One mg of the freeze dried NS was dissolved in an aqueous mucin solution (1 mg/mL) and left stirring, then centrifuged at 10,000 rpm for 1 min. The supernatant was collected for spectrophotometric analysis to determine the amount of free mucin, which was subtracted from the total amount of mucin added. All experiments were performed in triplicate.

### 4.8. Swelling Degree Evaluation

Dry β-CD and LC NSs of known weights were immersed in buffer solutions with different pH values at room temperature (i.e., pH 5.5 and pH 7.4 PBS). At pre-determined time intervals, the swollen NSs were centrifuged and removed from the buffer, surface blotted, and directly weighed. The procedure was repeated until there was no further weight increase. The swelling degree (SD) was then calculated as follows:

$$\mathrm{SD}=\frac{\left(\mathrm{Wt}\;-\;\mathrm{Wd}\;\right)}{\mathrm{Wd}}$$

where Wt is the weight of the swollen specimen and Wd is the weight of the specimen in the dry state. All experiments were performed in triplicate.

### 4.9. Viscosity Determination

The nanohydrogelsgels, either loaded or unloaded were characterized in vitro as aqueous nanosuspensions. The pH was recorded at room temperature using an Orion 420 A pH meter. The viscosity of the samples was evaluated using an Ubbelohde capillary viscometer (Schott Gerate, Mainz, Germany).

### 4.10. Rokitmycin Release Studies

In vitro release studies were carried out using multi-compartment rotating cells with a dialysis membrane (Sartorius, cut off 12,000 Da) placed between donor and receiving compartments. The donor phase consisted of RK-loaded NSs suspended in 1 ml of distilled water and the receiving phase phosphate buffer solutions with 0.1% sodium dodecyl sulfate at pH value of 5.5. The receiving phase was collected and replaced with fresh medium at fixed time and analyzed using the HPLC.

### 4.11. Mathmatical Models Fitting

Higuchi published probably the most famous and most often used mathematical equation to describe the release rate of drugs from matrix systems. Mathematical relationships developed by Higuchi are related to particles of active dispersed in homogeneous matrices submitted to a diffusing medium, where the amount of drug released is proportional to the square root of time [45]:

f1=Q=KH√t

Korsmeyer–Peppas power law model is useful for the study of drug release from polymeric systems when the release mechanism is not known or when more than one type of phenomenon of drug release is involved. It can be seen as a generalization of the observation of the superposition of two apparently independent mechanisms of drug transport, relaxation, and diffusion. Depending on the value of *n* that better adjusts to the release profile of an active agent in a matrix system [45].

F=(MtM)=Km · tn

### 4.12. Evaluation of Biocompatibility

Hemolysis assay was performed to evaluate the biocompatibility of the LC PYRO NSs, previous studies on β-CD PYRO NSs showed that it was biocompatible [13]. For hemolytic activity determination, 100 uL of different LC concentrations was incubated with 900 uL of blood diluted with NaCl (0.9% pH 7.4) at 37 °C for 90 min. After incubation, the samples were centrifuged at 1000 rpm for 5 min to separate the plasma. The amount of hemoglobin released due to hemolysis was determined spectrophotometrically (absorbance readout at 543 nm using a Beckman DU spectrophotometer). The hemolytic activity was calculated to compare with a negative control consisting of diluted blood without the addition of the samples. Complete hemolysis (positive control) was induced by the addition of Triton (20% *w*/*v*).

## Figures and Tables

**Table 1:** Physicochemical characterization of blank and RK-loaded NSs.

Sample	Blank β-CD PYRO	β-CD PYRO + RK	Blank LC PYRO	LC PYRO + RK
Average Diameter ± SD (nm)	215.6 ± 2.54	256.3 ± 13.8	213.5 ± 2.43	250.7 ± 11.0
PDI ± SD	0.021 ± 0.015	0.023 ± 0.013	0.048 ± 0.031	0.050 ± 0.010
Z-Potential ± SD (mV)	−28.45 ± 0.71	−26.89 ± 0.34	−27.15 ± 0.22	−25.73 ± 0.33
Mucoadhesion (%)	85.72	76.93	84.59	72.39

**Table 2:** Physicochemical characterization of blank and RK-loaded NSs after 1 month stored at 4 °C.

Sample	Blank β-CD PYRO	β-CD PYRO + RK	Blank LC PYRO	LC PYRO + RK
Average Diameter ± SD (nm)	223.6 ± 92.0	267.3 ± 14.5	223.5 ± 2.43	244.7 ± 25.2
PDI ± SD	0.024 ± 0.013	0.024 ± 0.013	0.028 ± 0.013	0.022 ± 0.015
Z-Potential ± SD (mV)	−28.38 ± 2.22	−27.86 ± 0.24	−26.15 ± 0.24	−27.44 ± 0.21

**Table 3:** Saturation solubility studies of the formulations with their enhancement factors.

Sample	RK	β-CD PYRO + RK	LC PYRO + RK	RK	β-CD PYRO + RK	LC PYRO + RK
pH	5.5	5.5	5.5	7.4	7.4	7.4
Solubility (μg/mL)	470.85	571.19	1455.13	450.44	525.21	1130.21
Enhancement Factor	-	1.21	3.09	-	1.17	2.51

**Table 4:** Swelling degree of nanohydrogels at different pH levels.

Sample	β-CD PYRO NS	LC PYRO NS	β-CD PYRO NS	LC PYRO NS
pH	5.5	5.5	7.4	7.4
Swelling Degree	684.90 ± 10.62	892.45 ± 7.96	1182.69 ± 11.67	1105.76 ± 10.94

**Table 5:** Mathematical models fitting of release profiles.

Sample	β-CD PYRO + RK (pH 5.5)	β-CD PYRO + RK (pH 7.4)	LC PYRO + RK (pH 5.5)	LC PYRO + RK (pH 7.4)
Higuchi Model (r^2^)	0.9957	0.9942	0.9951	0.9895
Korsmeyer–Peppas Model (r^2^)	0.9988	0.9781	0.9940	0.9756

## Data Availability

Not applicable.
